# Remarkable preservation of Ca^2+^ homeostasis and inhibition of apoptosis contribute to anti-muscle atrophy effect in hibernating Daurian ground squirrels

**DOI:** 10.1038/srep27020

**Published:** 2016-06-03

**Authors:** Weiwei Fu, Huanxin Hu, Kai Dang, Hui Chang, Bei Du, Xue Wu, Yunfang Gao

**Affiliations:** 1 Key Laboratory of Resource Biology and Biotechnology in Western China (Northwest University), Ministry of Education, Xi’an 710069, China; 2 National Research Center for Veterinary Medicine, Luo Yang 471003, China; 3 Shaanxi Institute of International Trade and Commerce, Xian Yang 712046, China; 4 Department of Burns and Cutaneous Surgery, Xijing Hospital, Fourth Military Medical University, Xi’an 710032, China

## Abstract

The underlying mechanisms that hibernators deviated from muscle atrophy during prolonged hibernating inactivity remain elusive. This study tested the hypothesis that the maintenance of intracellular Ca^2+^ homeostasis and inhibition of apoptosis would be responsible for preventing muscle atrophy in hibernating Daurian ground squirrels. The results showed that intracellular Ca^2+^ homeostasis was maintained in soleus and extensor digitorum longus (EDL) in hibernation and post-hibernation, while cytosolic Ca^2+^ was overloaded in gastrocnemius (GAS) in hibernation with a recovery in post-hibernation. The Ca^2+^ overload was also observed in interbout arousals in all three type muscles. Besides, the Bax/Bcl-2 ratio was unchanged in transcriptional level among pre-hibernation, hibernation and interbout arousals, and reduced to a minimum in post-hibernation. Furthermore, the Bax/Bcl-2 ratio in protein level was reduced in hibernation but recovered in interbout arousals. Although cytochrome C was increased in GAS and EDL in post-hibernation, no apoptosis was observed by TUNEL assay. These findings suggested that the intracellular Ca^2+^ homeostasis in hibernation might be regulated by the cytosolic Ca^2+^ overload during interbout arousals, which were likely responsible for preventing muscle atrophy via inhibition of apoptosis. Moreover, the muscle-specificity indicated that the different mechanisms against disuse-induced atrophy might be involved in different muscles in hibernation.

Calcium homeostasis is an important aspect to maintain intracellular environment in mammals. Under normal condition, intracellular Ca^2+^ concentration is accurately regulated to retain a steady level. However, skeletal muscle disuse (e.g. spaceflight, hind limb unloading and bed rest) leads to the disturbance of intracellular Ca^2+^ homeostasis, and mainly exhibits Ca^2+^ overload. For instance, the intracellular resting Ca^2+^ concentration was significantly elevated by 246% and 215% in rats’ soleus (SOL) muscle and gastrocnemius (GAS) muscle, respectively, after 4 weeks of hindlimb immobilization^1^. Meanwhile, the significant increase in intracellular Ca^2+^ level was also reported in SOL muscle of hindlimb unloading rats^2^. Similarly, our previous study had reported that the intracellular resting Ca^2+^ concentration was increased by 330% in SOL muscle of rats after 14 days of hindlimb suspension^3^. The SOL muscle contains large amounts of slow type fibers (more than 84%), while the portion of fast type fibers in EDL is no more than 12%^4^,^5^. These findings showed that the slow-twitch muscles (SOL) were more sensitive to disuse than the fast-twitch muscles (EDL) and mixture muscles (GAS), which was closely associated with the higher Ca^2+^ sensibility in slow type fibers^6^,^7^.

Intracellular Ca^2+^ overload plays an important role in the mechanisms of disuse-induced muscle atrophy. Calcium-activated proteases (calpains), which can be activated by elevated intracellular Ca^2+^ concentration, contributed to the degradation of filaments and initiation of most proteolytic pathways (e.g. the ubiquitin–proteasome pathway) in disuse^8^,^9^. Increased protein degradation triggered by calpains is considered as one of the most important pathways in disuse-induced muscle atrophy^10^. In addition, apoptosis has been demonstrated as a key mechanism in the disuse-induced muscle atrophy^11^,^12^. Cytosolic Ca^2+^ overload is associated with mitochondrial apoptosis and have been found in the early steps of apoptosis^13^. The Ca^2+^ uptake by mitochondria might be involved in the mechanism of apoptosis, in which the increase of cytosolic Ca^2+^ can promote Ca^2+^ accumulation in mitochondria and trigger mitochondrial depolarization while the concentration of mitochondrial Ca^2+^ was up to the threshold value^14^. Subsequently, the pro-apoptotic protein Bax was activated, translocated and inserted into the outer membrane of mitochondria via forming Bax/Bax-homo-oligomerization^15^. This was followed rapidly by the formation and opening of a mitochondrial permeability transition pore (mPTP), through which the cytochrome C, a mitochondria-resided apoptogenic factor, was released into the cytosol and eventually led to the cleavage of nuclear DNA and cell apoptosis^16^. The DNA fragmentation was reported as an indicator of apoptosis in the early^12^,^17^ or late phases^18^ in hindlimb unloading animals. Furthermore, Bcl-2, another Bcl-2 family protein, inhibited apoptosis via suppression of the formation of Bax/Bax-homo-oligomerization^19^. The ratio of Bax/Bcl-2 serves as a molecular indicator in determining whether cells will undergo apoptosis^20^, which showed a significant rise in SOL muscle after 6 hours hindlimb unloading as compared with control mice^12^.

Hibernators like Daurian ground squirrels (*Spermophilus dauricus*) appear to deviate from significant loss of myofibrillar contents and force of skeletal muscles during long period of hibernation inactivity, which provide a useful model to study mechanisms of preventing disuse-induced skeletal muscle atrophy^21^,^22^,^23^,^24^. Mammalian hibernators appear to preserve the slow-twitch muscles^23^,^24^. The response of hibernators to hindlimb unloading is quite different from non-hibernators in which the loss of mass and force in slow-twitch muscles is more severe than that in fast-twitch muscles. Moreover, a shift from fast type fibers to slow type fibers was found mainly in fast-twitch muscles of mammalian hibernators^21^,^23^,^25^. It seemed that different types of muscles showed different degrees of Ca^2+^ overload in disuse-induced atrophy. Based on these findings, we hypothesized that intracellular Ca^2+^ was involved in the mechanisms of protecting from muscle atrophy in hibernators. However, the research on intracellular Ca^2+^ is quite rare and changes of intracellular Ca^2+^ level in different type of muscles in mammalian hibernators are still unclear. We hypothesized that hibernators would be able to prevent Ca^2+^ overload, keep intracellular Ca^2+^ level steady and avoid initiation of apoptosis in months of hibernation. We also hypothesized that the muscle type might be an important impact factor on intracellular Ca^2+^ level. Based on these hypotheses, we examined intracellular Ca^2+^ concentration, expressions of apoptosis-related factors (including Bax, Bcl-2 and cytochrome C) and DNA fragmentation in different types of skeletal muscles in Daurian ground squirrels, and further explored the molecular mechanism involved in preventing muscle atrophy in mammalian hibernators.

## Results

### Body weight, muscle wet weight and muscle wet weight/body weight ratio

SOL, GAS and EDL muscles in experimental animals have been used extensively in skeletal muscle research. GAS muscle wet weight was 19% lower (*P* = 0.021) in post-hibernation (POST) than in pre-hibernation (PRE) squirrels, while SOL (−9%, *P* = 0.21) and EDL (−16%, *P* = 0.14) muscles did not change (Table 1). Moreover, the ratio of muscle wet weight/body weight in GAS was 25% higher (*P* = 0.02) in hibernation(HIB) and 27% higher (*P* = 0.006) in interbout arousals (IBA) than in PRE group. The ratios of muscle wet weight/body weight in EDL and SOL showed a 28–34% increase (*P* < 0.01) in HIB, IBA and POST groups as compared with PRE group (Table 1).

### Muscle fiber cross-sectional area (CSA) and muscle fiber type composition

**Figure 1:**
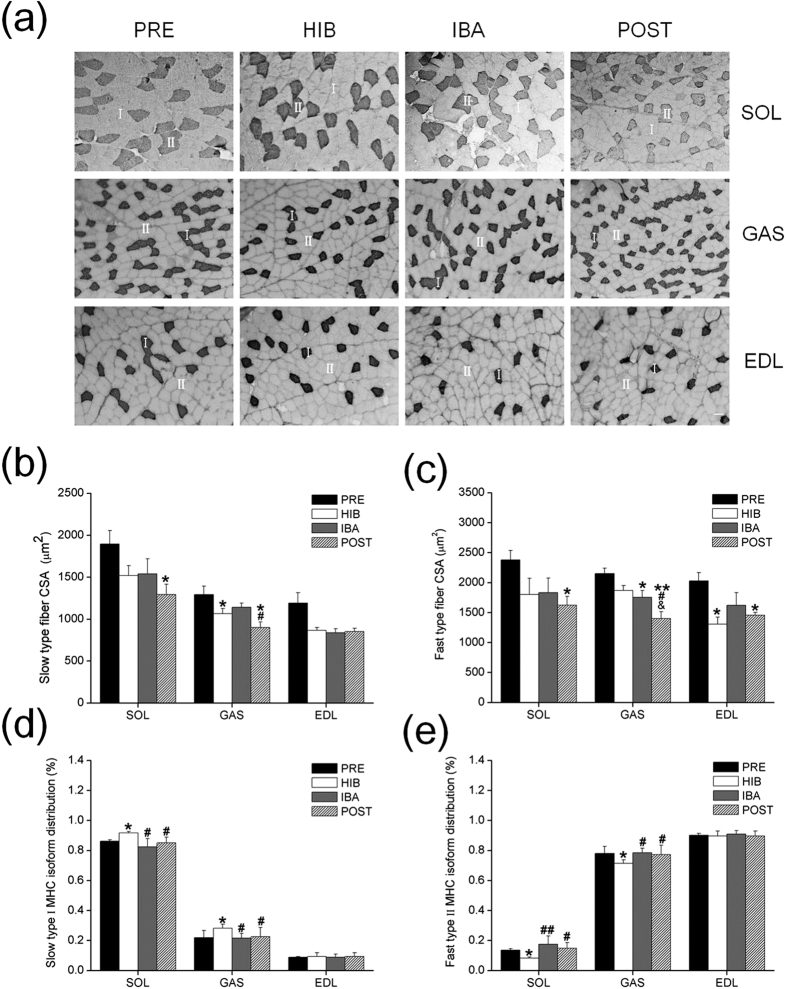
Measurements of muscle fiber in cross-sectional area (CSA) and MHC isoform distribution. **(a)** Representative immunohischemistry images of MHC I fibers (dark) in GAS and EDL muscles, and MHC II fibers (dark) in SOL muscle during four hibernation periods. I = type I MHC, II = type II MHC. Scale bar = 50 μm. **(b)** Bar graph depicting the changes in slow type fiber CSA. **(c)** Bar graph depicting the changes in fast type fiber CSA. **(d)** Percentage of the slow type I MHC isoform distribution. **(e)** Percentage of the fast type II MHC isoform distribution. SOL, **s**oleus; GAS, gastrocnemius; EDL, extensor digitorum longus; PRE, pre-hibernation; HIB, hibernation; IBA, interbout arousals; POST, post-hibernation. Values are represented as mean ± SE, n = 6. *P < 0.05 compared with PRE; **P < 0.01 compared with PRE; ^#^*P* < 0.05 compared with HIB; ^##^*P* < 0.01 compared with HIB; ^&^*P* < 0.05 compared with IBA.

Immunohischemistry analysis was used to the determination of different muscle fiber types (Fig. 1a). Fiber CSA in SOL was 31–32% (*P* < 0.05) reduced in POST in both slow (MHC I) and fast (MHC II) fibers as compared with PRE group, while no significant decrease in fiber CSA was observed in slow fiber in EDL, despite fiber CSA in fast fiber was 28–36% smaller (*P* < 0.05) in both HIB and POST. Compared with PRE group, fiber CSA in slow fiber of GAS was significantly reduced by 18% (*P* < 0.05) in HIB and 30% (*P* < 0.05) in POST, while CSA in fast fiber of GAS was significantly reduced by 18% (*P* < 0.05) in IBA and 35% (*P* < 0.01) in POST group. Moreover, fiber CAS in GAS showed a 21% decrease (*P* < 0.05) in slow fiber and 20% decrease (*P* < 0.05) in fast fiber in POST as compared with IBA squirrels. Significant decrease of fiber CSA in POST group was also observed in fast fiber of GAS as compared with IBA group (−19%, *P* < 0.05) (Fig. 1b,c).

The percentage of slow type fiber was higher (8% in SOL and 26% in GAS respectively, *P* < 0.05) in HIB than that in PRE and recovered to the level of PRE in both IBA and POST groups. In contrary, the percentage of fast type fiber was reduced significantly (40% in SOL and 9% in GAS respectively, *P* < 0.05) in HIB as compared with PRE group, and subsequently recovered to the level of PRE in both IBA and POST groups. However, the shift of fast-to-slow fibers was not observed in EDL (shown in Fig. 1d,e).

### Cytoplasm calcium concentration in single fiber

**Figure 2:**
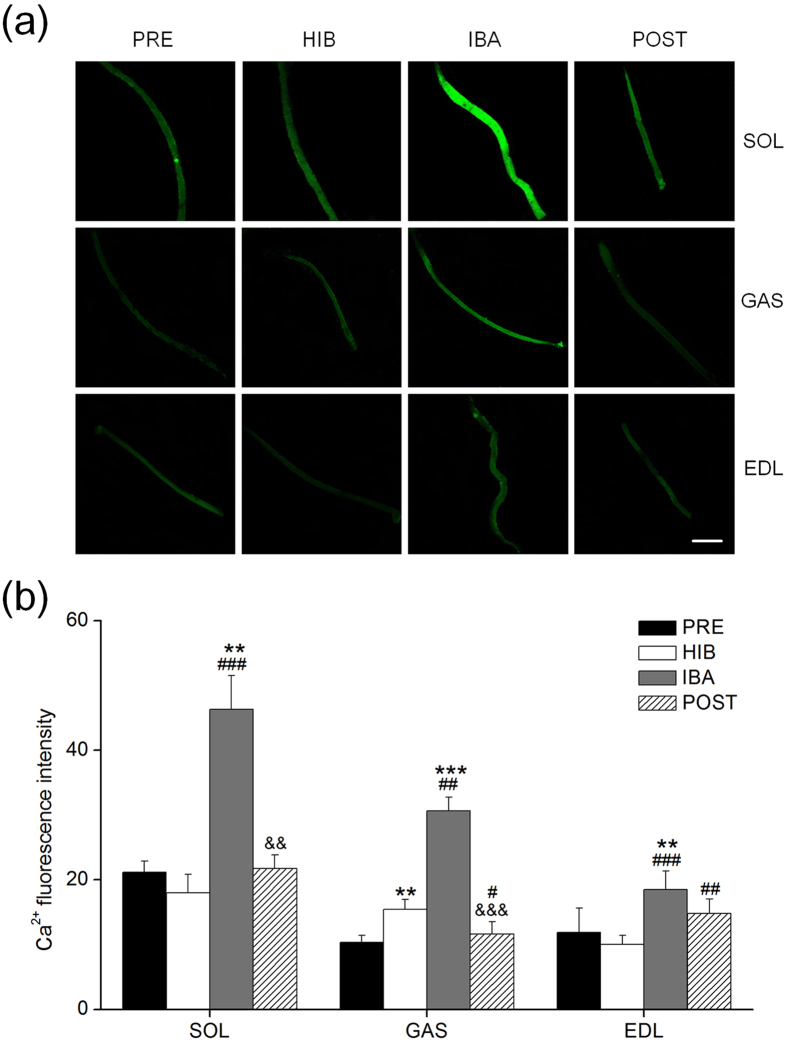
Change of intracellular Ca^2+^ fluorescence intensity of single muscle fiber in three different types of muscles during four hibernation periods. (**a**) Representative fluorescence images of single muscle fiber loaded by fluo-3/AM. Scale bar = 180 μm. (**b**) Bar graph depicting the changes in the mean intensity of intracellular Ca^2+^ fluorescence. Eight muscle fiber cells were analyzed in each group. Values are mean ± SE. **P < 0.01 compared with PRE; ***P < 0.001 compared with PRE; ^#^*P* < 0.05 compared with HIB; ^##^*P* < 0.01 compared with HIB; ^###^*P* < 0.001 compared with HIB; ^&&^*P* < 0.01 compared with IBA; ^&&&^*P* < 0.001 compared with IBA.

Figure 2a showed a series of two-dimensional confocal images of muscle fibers from all three muscles in PRE, HIB, IBA and POST groups. The muscle fibers exhibited a quite uniform green fluorescence, which was distributed mainly in the cytoplasm. The cytosolic Ca^2+^ fluorescence in GAS was significantly increased (49%, *P* = 0.004) in HIB group as compared with PRE group. It is noteworthy that the Ca^2+^ fluorescence in IBA group showed a notable increase (at least *P* < 0.01) in SOL (119%), GAS (156%) and EDL (56%) as compared with PRE group. However, the Ca^2+^ fluorescence in POST reduced significantly in SOL (53%, *P* = 0.001) and GAS (62%, *P* = 0.000) as compared to IBA group. Besides, the Ca^2+^ fluorescence in POST decreased significantly (25%, *P* = 0.03) in GAS but increased significantly (48%, *P* = 0.002) in EDL as compared to HIB group (Fig. 2b).

### Relative mRNA expression of *bax* and *bcl-2*

**Figure 3:**
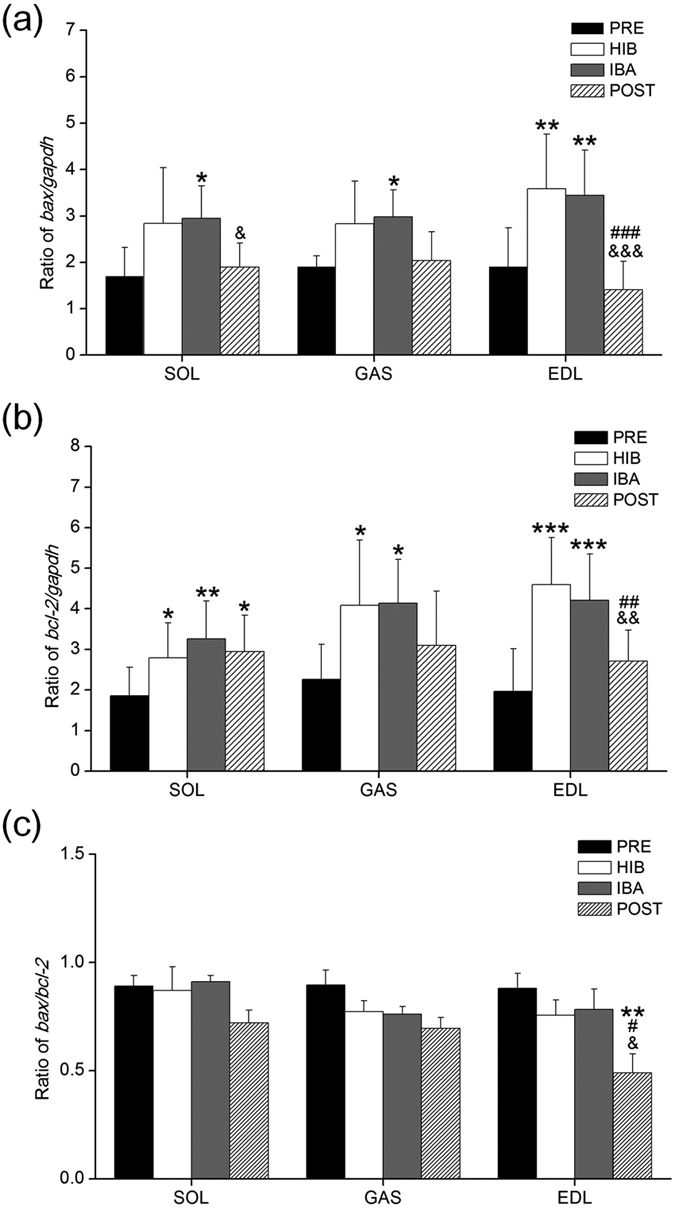
Changes in the transcript levels of *bax*, *bcl-2* and the ratio of *bax/bcl-2* in three different types of muscles during four hibernation periods. (**a**) The ratio of *bax/gapdh*. (**b**) The ratio of *bcl-2/gapdh.* (**c**) The ratio of *bax* to *bcl-2*. Values are mean ± SE, n = 6. *P < 0.05 compared with PRE; **P < 0.01 compared with PRE; ***P < 0.001compared with PRE; ^#^*P* < 0.05 compared with HIB; ^##^*P* < 0.01 compared with HIB; ^###^*P* < 0.001 compared with HIB; ^&^*P* < 0.05 compared with IBA; ^&&^*P* < 0.01 compared with IBA; ^&&&^*P* < 0.001 compared with IBA.

Relative experissions of *bax and bcl-2* in mRNA transcript level were showed in Fig. 3a,b respectively. The *bax* mRNA transcript level in EDL (88%, *P* < 0.01) and *bcl-2* mRNA transcript level in SOL (50%, P < 0.05) and EDL (135%, P < 0.001) were higher in HIB than in PRE. All three different types of muscles showed a 52–80% higher level of *bax* mRNA transcript and a 75–115% higher level of *bcl-2* mRNA transcript (at least *P* < 0.05) in IBA, respectively, as compared with PRE. Additionally, significant difference in POST squirrels was not found in GAS, but in EDL there was a dramatic reduction in *bax* mRNA transcript level (59–61%, *P* < 0.001) and *bcl-2* mRNA transcript level (36–41%, *P* < 0.01), respectively, as compared with HIB and IBA squirrels.

The ratio of *bax/bcl-2*, an important indicator of apoptosis, was analyzed in GAS, SOL and EDL muscles. The ratio remained constant in SOL and GAS muscle in four hibernation groups. A significant difference in *bax/bcl-2* ratio was observed in EDL of POST group as compared to PRE (−48%, *P* < 0.01), HIB (−38%, *P* < 0.05) and IBA (−43%, *P* < 0.01) squirrels (Fig. 3c).

### Relative protein expression of Bax, Bcl-2 and cytochrome C

**Figure 4:**
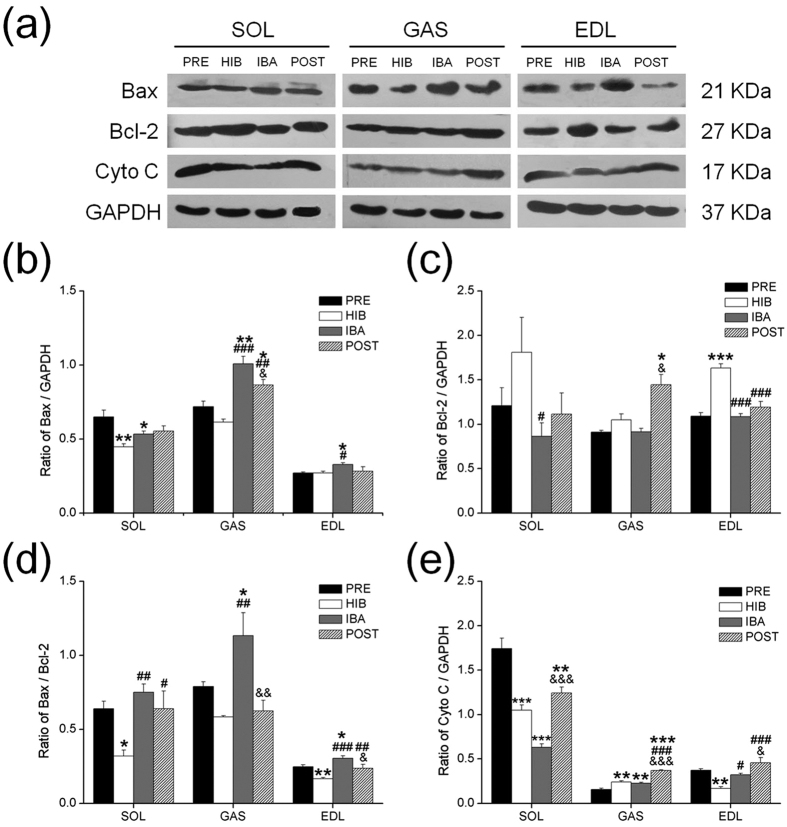
Changes in the protein levels of Bax, Bcl-2 and cytochrome C (Cyto C) and the ratios of Bax/Bcl-2 in three different types of muscles during four hibernation periods. (**a**) Representative immunoblots of Bax, Bcl-2 and cytochrome C in three different types of muscles during four hibernation periods. (**b**) The Ratio of Bax to GAPDH. (**c**) The Ratio of Bcl-2 to GAPDH. (**d**) The ratio of Bax to Bcl-2. (**e**) The Ratio of Cyto C to GAPDH. Values are represented as mean ± SE, n = 6. *P < 0.05 compared with PRE; **P < 0.01 compared with PRE; ***P < 0.001 compared with PRE; ^#^*P* < 0.05 compared with HIB; ^##^*P* < 0.01 compared with HIB; ^###^*P* < 0.001 compared with HIB; ^&^*P* < 0.05 compared with IBA; ^&&^*P* < 0.01 compared with IBA; ^&&&^*P* < 0.001 compared with IBA.

The contents of Bax, Bcl-2 and cytochrome C proteins were detected by western blot analysis as shown in Fig. 4a. The Bax expression in SOL was significantly reduced by 31% in HIB (*P* < 0.01) and 18% in IBA (*P* < 0.05), respectively, as compared with PRE squirrels. The Bax expression in IBA squirrels was 40% higher (*P* < 0.01) than that in PRE squirrels and was 63% higher (*P* < 0.01) than that in HIB squirrels; meanwhile, the Bax expression in POST squirrels was 20% higher (*P* < 0.05) than that in PRE squirrels and was 41% higher (*P* < 0.01) than that in PRE in GAS. Moreover, the Bax expression was 14% lower (*P* < 0.05) in POST squirrels than that in IBA squirrels. The Bax expression in EDL in IBA squirrels was 22% higher than that in PRE squirrels (*P* < 0.05) and 21% higher than that in HIB squirrels (*P* < 0.05) (Fig. 4b).

The Bcl-2 protein level was significantly reduced by 51% (*P* < 0.05) in SOL in IBA squirrels as compared with HIB squirrels, while it was significantly increased in a similar extent (58%, *P* < 0.05) in GAS in POST squirrels as compared with both PRE and IBA squirrels. However, the Bcl-2 protein level changed notably in EDL. In HIB squirrels, Bcl-2 protein level in EDL was significantly increased by 50% as compared with PRE squirrels (*P* < 0.001), but reduced significantly by 50% in IBA squirrels (*P* < 0.001) and 37% in POST squirrels (*P* < 0.001), respectively, as compared with HIB squirrels (Fig. 4c).

The ratio of Bax/Bcl-2 in SOL showed a significant reduction in HIB (−50%, *P* < 0.05) as compared with PRE group, while it was increased in both IBA (57%, *P* < 0.01) and POST (50%, *P* < 0.05) groups as compared with HIB group. The ratio of Bax/Bcl-2 in GAS in IBA group was 81% higher (*P* < 0.05) than that in PRE and 93% higher (*P* < 0.01) than that in HIB, however, it was significantly reduced by 45% (*P* < 0.01) in POST than that in IBA group. Compared with PRE group, the ratio of Bax/Bcl-2 in EDL was significantly reduced by 32% (*P* < 0.01) in HIB, but increased by 20% (*P* < 0.05) in IBA. Moreover, the ratio of Bax/Bcl-2 in EDL was significantly increased by 74% in IBA (*P* < 0.001) and 41% in POST (*P* < 0.01) as compared with HIB group, while it was significantly reduced by 20% (*P* < 0.05) in POST as compared with IBA group (Fig. 4d).

The level of cytochrome C expression in SOL was significantly reduced by 40% in HIB (*P* < 0.001), 63% in IBA (*P* < 0.001) and 29% in POST (*P* < 0.01), respectively, as compared with that in PRE group, but it was significant increased by 96% in POST (*P* < 0.001) as compared with IBA group. A notable increased level of cytochrome C was observed in GAS in POST group as compared with PRE (123%, *P* < 0.001), HIB (53%, *P* < 0.001) and IBA (61%, *P* < 0.001). The level of cytochrome C in EDL was significantly reduced by 55% in HIB as compared with PRE, but increased in IBA. Moreover, the cytochrome C in EDL in POST group showed a 169% higher level (*P* < 0.001) than that in HIB, and a 41% higher level (*P* < 0.05) than that in IBA (Fig. 4e).

### DNA fragmentation

**Figure 5:**
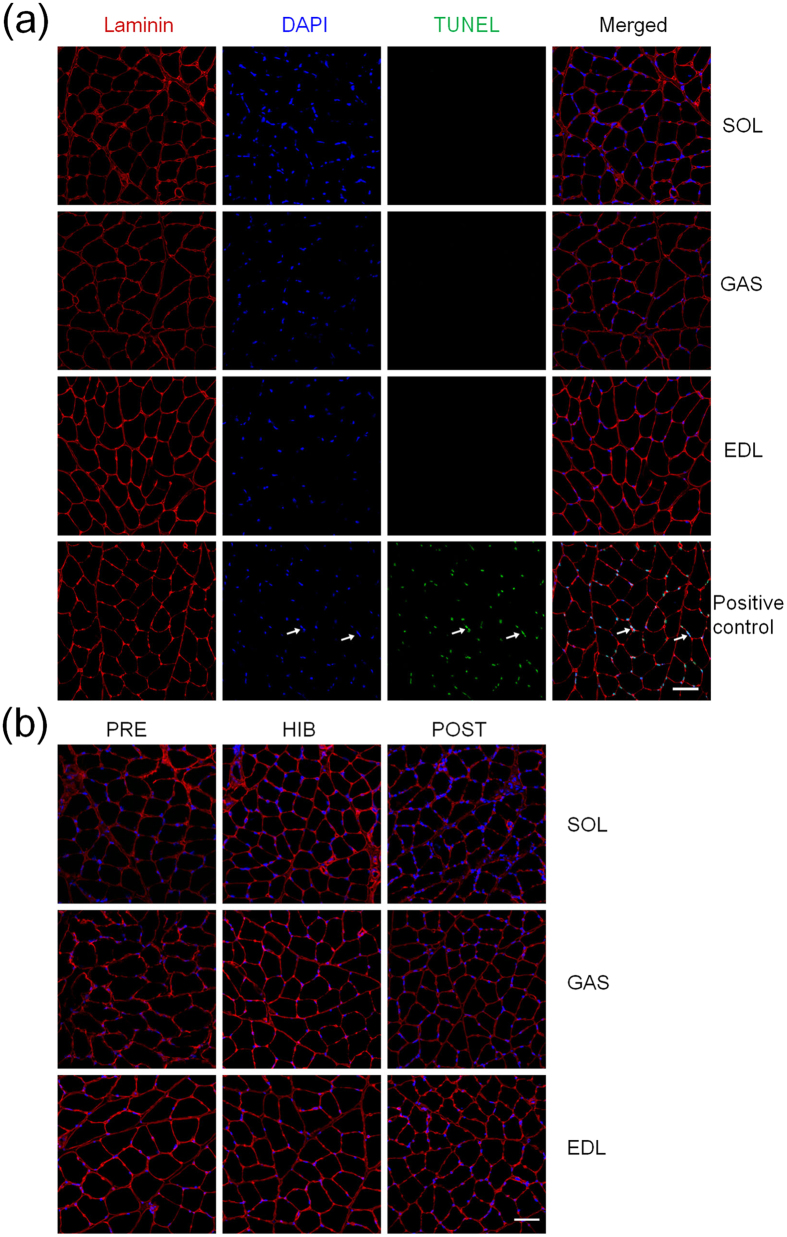
Fluorescent terminal deoxynucleotidyl transferase biotin-dUTP nick end labeling (TUNEL) stain in three different types of muscles during four hibernation periods. (**a**) Representative images showing cross-sections of SOL, GAS and EDL muscles from IBA squirrels and positive control for TUNEL labeling (green, arrows) counterstained with DAPI (blue) for nuclei identification. Red represents laminin stain of myofiber interstitial tissue. (**b**) Representative images showing cross-sections of SOL, GAS and EDL muscles from PRE, HIB and POST squirrels with TUNEL labeling. Scale bar = 50 μm.

TUNEL staining provided a direct evidence of apoptosis. As we hypothesized, no TUNEL-positive nuclei was observed in all three types of muscles in IBA group (Fig. 5a), although a striking Ca^2+^ overload was shown in IBA. Moreover, there was also no TUNEL-positive nuclei observed in other three groups as shown in Fig. 5b.

## Discussion

Despite experiencing months of hibernation, most skeletal muscles in ground squirrel maintained steady wet weight and muscle fiber type composition, which was opposite with non-hibernators following disuse. Meanwhile, a shift of fast-to-slow fibers was observed in SOL and GAS muscles in hibernation squirrels and the ratio of muscle wet weight/body weight was increased in all three types of muscles, which were contrary to non-hibernator following hindlimb unloading^26^,^27^. However, except in slow fibers of EDL muscle, muscle fiber CSA showed a reduction in both slow and fast fibers of three muscle types, which was similar with non-hibernators induced by one or more weeks of hindlimb unloading^28^,^29^. Although muscle fiber CSA showed a significant reduction, the data from muscle wet weight, muscle fiber type composition and ratios of muscle wet weight/body weight all supported the view that hibernators can protect skeletal muscle from disuse-induced atrophy^21^,^30^,^31^. More importantly, not all types of muscles could avoid muscle atrophy after prolonged inactivity in hibernation, and the response of different types of muscles to hibernation seemed irrelevant to the fiber types, which was in consistent with previous studies^21^,^23^,^25^. For example, GAS (dominantly composed of mixture fibers) showed a higher loss of muscle mass and a more severe reduction of fiber CSA in both slow and fast fibers than those in the slow-twitch SOL muscle and fast-twitch EDL muscle. However, it remains unclear whether different muscle types have any effects on intracellular Ca^2+^ concentration and cell apoptosis in hibernating ground squirrels.

The present study showed that the SOL and EDL muscles still maintained a steady Ca^2+^ level after months of hibernation as similar as the PRE group. Obviously, hibernating squirrels were able to maintain intracellular Ca^2+^ homeostasis and recover from the disorder of intracellular Ca^2+^ homeostasis, which was in consistent with our previous report that the protein expressions and activities of calpains had no significant changes in hibernation and post-hibernation^32^. Other studies also reported that protein degradation was inhibited in hibernating mammals^33^,^34^,^35^.

It is noteworthy that the different responses of muscle types were also reflected in intracellular Ca^2+^ concentration in hibernating squirrels. In contrast with SOL and EDL, Ca^2+^ concentration in GAS was significantly increased during hibernation as compared with PRE group, which may activate calpains and promote protein degradation during hibernation. Skeletal muscle is the largest protein storage tissue and the most important source of gluconeogenic precursors during prolonged fasting in normothermic mammals^36^. Hibernators experienced fasting for a long time and muscle protein degradation might be involved in energy provision during hibernation. As shown in Table 1 and Fig. 1, GAS, but not SOL and EDL, was more sensitive to hibernation inactivity and degraded selectively, which might be due to that GAS is one of the largest muscles in hindlimb muscles and the influence of GAS degradation on individual movement function is relatively small. Moreover, we also found that the percentage of increased intracellular Ca^2+^ concentration in GAS was lower than that in non-hibernators exposed to hindlimb unloading^1^, which might explain why the hibernators only showed limited loss of GAS muscle relative to the higher muscle loss induced by hindlimb unloading in rats. Besides, the intracellular Ca^2+^ level can keep steady or recover from the disorder of Ca^2+^ homeostasis in post-hibernation, which was also in contrast with non-hibernators. The differences between hibernators and non-hibernators indicate that the maintenance of intracellular Ca^2+^ homeostasis is an important mechanism involved in preventing muscle atrophy in hibernators.

Another important finding in this study was a striking increase in intracellular Ca^2+^ concentration in interbout arousal squirrels. During interbout arousals, intracellular Ca^2+^ concentration in single fiber was significantly increased in SOL, GAS and EDL muscles as compared with pre-hibernation. In non-hibernators, the intracellular Ca^2+^ overload was observed after 10–14 days hindlimb unloading^2^,^37^. However, squirrels showed only a short period of Ca^2+^ overload during interbout arousals^38^,^39^ and Ca^2+^ overload was reserved partially (in GAS) or completely (in the SOL and EDL) when animals entered torpor again. The results suggested that the interbout arousals might play an important role against disuse-induced muscle atrophy through regulation of intracellular Ca^2+^ level in hibernating mammals. The interbout arousal is known as a periodic euthermic arousal interspersed in months of hibernation in which the body temperature of hibernators returned to normal temperature from hypothermia, accompanied with a high metabolic rate, and the greatest part of all of the energy expended during the hibernation season is required for interbout arousals^40^,^41^. The mechanism of the elevated intracellular Ca^2+^ content in muscle fibers is still unclear and future studies will be required to determine the cellular and molecular mechanisms of cytosolic excessive Ca^2+^ in skeletal muscles of hibernators.

Additionally, the rise of intracellular Ca^2+^ might activate calpains, which led to protein degradation, thereby producing enough energy to rapidly elevate body temperature in the phase of awakening and maintain the normal body temperature during interbout arousals. Energy production in interbout arousal is increased because of muscle protein degradation, which is consistent with the previous report that glucose supplements lipid fuels during awakening, and carbohydrates are an important fuel for shivering thermogenesis^42^. Also, tissue protein is degraded to supply the gluconeogenic demand during interbout arousals because of the shortage of glycogen stores and increased demand for glucose^35^. However, we found that the loss of muscle mass induced by protein degradation become significant after squirrels experiencing more interbout arousals. This is the reason why the significant reduction in the loss of muscle mass in GAS muscle was only found in POST group (more than three months of hibernation), rather than in IBA and HIB group (two months of hibernation). Therefore, we hypothesized that hibernators with more periodic arousals would result in more degradation of muscle proteins, which indicated that the number of interbout arousals was correlated positively with the degree of muscle atrophy. But the relationship between interbout arousals and muscle atrophy needs further investigations.

Although the research investigated apoptosis after disuse in hindlimb skeletal muscles was increased^18^,^43^,^44^, limited data showed in skeletal muscles of hibernators^45^. It had been reported that no apoptotic-related proteins (including Bcl-2, p-Bcl-2 T56, p-Bcl-2 S70, Bcl-xL, Mcl-1, BI-1, and cIAP1/2) had changed in mixture skeletal muscle of ground squirrels in hibernation except xIAP, which was significantly increased^45^. The research also indicated the inhibition of apoptosis during torpor. In the present study, protein levels of Bax, Bcl-2 and cytochrome C and DNA fragmentation were determined to evaluate apoptosis in different muscles. DNA fragmentation is one of the hallmark morphological features of apoptosis. The results from TUNEL detection showed no DNA fragmentation in three types of muscles during four hibernation periods, which provided a direct evidence of apoptosis inhibition in hibernators. Although no apoptosis was observed, a seris of important apoptotic index (Bax, Bcl-2, ratio of Bax/Bcl-2 and cytochrome C) showed significant changes in different hibernation periods and different types of muscles.

In our study, the Bax was significantly increased in interbout arousals in three types of muscles, and recovered to the level of pre-hibernation in post-hibernation group in both SOL and EDL muscles. In GAS muscle, Bax protein level still maintained in a higher level in post-hibernation than that in pre-hibernation and hibernation. The level of Bcl-2 protein in interbout arousals was significantly reduced in SOL and EDL muscles as compared with hibernation group, whereas no significant difference was observed in interbout arousals in all three types of muscles as compared with pre-hibernation. The ratio of Bax/Bcl-2 showed a significant reduction in hibernation (in SOL and EDL muscles) as compared with pre-hibernation group and a significant increase in interbout arousals (in three types of muscles) as compared with hibernation group. The data of Bax protein content and ratio of Bax/Bcl-2 were in consistent with the intracellular Ca^2+^ concentration exhibiting a significant increase in interbout arousals as compared with hibernation group, which indicated the regulation of apoptosis by intracellular Ca^2+^. Importantly, the above results suggested that apoptosis was inhibited in hibernation, although the apoptosis inhibition was attenuated in interbout arousals. Moreover, the ratio of Bax/Bcl-2 in all three types of muscles were unchanged in post-hibernation group compared with pre-hibernation group, which indicated that the apoptosis was not induced after months of hibernating inactivity^45^. In addition, we also found that the level of cytochrome C protein was significantly increased in GAS and EDL muscles in post-hibernation in the study. The level of cytochrome C protein in different types of muscles was in consistent with the previous report in which the hibernators preserved the slow-twitch muscles^23^,^24^. The release of cytochrome C from mitochondria can be triggered by several factors in the mitochondrial-mediated apoptosis pathway^16^. However, the increase of cytochrome C was not enough to trigger apoptosis as shown in Fig. 5. Thus, skeletal muscles in hibernators appeared to possess a resistance to apoptosis during months of inactivity.

Previous studies on arctic ground squirrels reported that the expression profiles of apoptosis-related genes increased significantly in skeletal muscle during arousal phases^46^. Similarly, *bax* and *bcl-2* mRNA transcript in three types of muscles also showed a significant increase in interbout arousals in our study. Transcription is inhibited in hibernation and recovered during interbout arousals in hibernators’ tissue cells^47^,^48^,^49^. However, *bax* mRNA transcript in EDL was increased in hibernation, while *bcl-2* mRNA transcript in all three types of muscles in hibernation was increased as well as in interbout arousals, which indicated that *bax* and *bcl-2* might be involved in the regulation of mitochondria-mediated apoptosis in hibernating ground squirrels. Additionally, the ratio of *bax/bcl-2*, as an indicator of apoptosis, decreased to a minimum level in all three muscle types in post-hibernation. Together, these data indicated that the anti-apoptosis function might initiate early in the cellular transcriptional level.

In summary, our data support an essential role of steadiness of intracellular Ca^2+^ concentration in single fiber in the mechanism against disuse-induced muscle atrophy through inhibiting cell apoptosis in three different types of muscles of ground squirrels in hibernation and post-hibernation. The Ca^2+^ overload in interbout arousals might be the energy supply during interbout arousals and regulation of recovery of intracellular Ca^2+^ homeostasis in hibernation. In addition, although the level of cytochrome C showed a significant increase in post-hibernation squirrels, no apoptosis was observed in the whole hibernation period. Furthermore, our data provide a novel mechanistic link between muscle-specificity, not fiber-specificity, and some relevant indexes of muscles including the loss of muscle mass, the transient of muscle fiber types, the cytosolic Ca^2+^ overload and the protein level of Bax, which exhibited different changes in SOL, EDL and GAS muscles in hibernation. Our study demonstrated that there might exist an intrinsic mechanism for protecting from muscle atrophy despite experiencing months of hibernating inactivity in ground squirrels, which was mediated by the inhibition of cytosolic Ca^2+^ overload and cell apoptosis. Our study also pointed out the importance of muscle types to fully understand the molecular mechanisms against disuse-induced atrophy in hibernation.

## Methods

### Animals and groups

All procedures were approved by the Laboratory Animal Care Committee of theP. R. China Ministry of Health. The ground squirrels were prepared as previously described by our laboratory^21^,^32^. Briefly, thirty-two Daurian ground squirrels of both sexes were caught from the Weinan region in the Shaanxi Province of China and provided water and rat chow *ad libitum*. When squirrels gradually entered torpor at the beginning of November, they were transferred to a cold room at 4–6 °C. The dates of entering torpor were determined by putting sawdust on the back of each subject and reaching a body temperature (T_b_) below 9 °C. Body temperature was determined by thermal imaging using a visual thermometer (Fluke VT04 Visual IR Thermometer, USA). Once squirrels entered torpor, food and water were removed and daily observations were made during the experimental period. Based on our year-by-year records, most animals return to hibernation after 1 to 2 days of interbout arousals. Animals that aroused stayed awake for more than two days and were assigned to POST.

Animals were randomly divided into four groups (n = 8): (1) PRE: no hibernation animals investigated in late-autumn and maintaining a T_b_ of 36–38 °C; (2) HIB: animals after two months of hibernation with T_b_ maintained at 5–8 °C; (3) IBA: awake animals after two months of hibernation with T_b_ returned to 34–37 °C for several hours; (4) POST: animals awaking from hibernation and maintaining a T_b_ of 36–38 °C for more than 2 days in March of next year.

### Isolation of single muscle fiber

Animals were deeply anaesthetized with 90 mg/kg sodium pentobarbital i.p. The muscle sample with tendon was dissected gently free of surrounding tissues and myolemma, leaving its blood and nerve supply intact. Clamped the tendon with a tip tweezers along the longitudinal axis of the muscle to tear it into two full length of the muscle strips (avoid tearing). Then the muscles were washed with 20 mL of phosphate-buffered saline (PBS, 137 mM Sodium Chloride, 2.7 mM Potassium Chloride, 4.3 mM Disodium Chloride, 1.4 mM Monopotassium Phosphate, pH 7.4) and acutely dissociated by 3 mL enzymatic digestion solution containing 0.35% collagenase I (Sigma-Aldrich, Saint Quentin Fallavier, France) plus 0.17% neutral protease (Sigma-Aldrich) incubated at 33 °C on an orbital shaker for 2 h. The enzymatic digestion solution was saturated with 95% O_2_ and 5% CO_2_ gas mixture, so that the muscle fibers could be completely digested. Finally, the digestion solution was washed out with PBS solution and the muscles were agitated gently and repeatedly with pipettes. The dissociated single muscle fiber was platted on culture chamber slides and observed under the inverted microscope (Olympus, IX2-ILL100, Japan).

Muscle samples for other experiments were subsequently stored in liquid nitrogen until further processing. At the end of surgical intervention, the animals were sacrificed by an overdose injection of sodium pentobarbital. The Northwest University Ethics Committee reviewed and approved all procedures in the animal studies. All procedures were carried out in accordance with the approved guidelines.

### Measurement of intracellular calcium

Fluo-3-acetoxymethylester (Fluo-3/AM) (Invitrogen, Carlsbad, USA), which exhibited an increase in fluorescence upon binding Ca^2+^, was used to indicate cytosolic free Ca^2+^ as previous described^50^. Briefly, the single muscle fibers were incubated with Fluo-3/AM in a concentration of 5 mM for 30 min at 37 °C. After incubation, the Fluo-3/AM-loaded muscle fibers on a glass slide were washed with fresh PBS and then scanned under a laser confocal microscope equipped with the Olympus FV10-ASW system (Olympus, FV10-MCPSU, Japan) by illuminating with a krypton/argon laser at 488 nm emitted light and capturing the emitting fluorescence at 526 nm. The fluorescence intensity was used to indicate the change of intracellular Ca^2+^ in muscle fibers. Quantification analysis of the fluorescence intensity was performed with the NIH Image software (Image-proplus 6.0).

### Immunohistochemistry analysis

Ten-μm thick frozen muscle cross-sections were cut from the mid-belly of each muscle at −20 °C with a cryostat (Leica, Wetzlar, CM1850, Germany), and stored at −80 °C for further staining. Immunohistochemistry was used to determine muscle fiber cross-sectional area and fiber type composition. The sections were air dried for 10 min and fixed in 4% paraformaldehyde in PBS (pH 7.4) for 20 min. Then sections were incubated in a blocking solution (5% BSA) (Boster, Wuhan, China) for 30 min at room temperature and in turn incubated in a primary antibody solution at 4 °C overnight. The primary antibodies included an anti-skeletal fast myosin antibody (Sigma, St. Louis, MO) to visualize the type II myosin heavy-chain (MHC) in SOL muscle, and an anti-skeletal slow myosin antibody (Sigma) to visualize the type I MHC in both GAS and EDL muscles. Subsequently, sections were placed in goat anti-mouse IgG (Boster) for 30 min, in SABC (Boster) for 30 min and in DAB (Boster) for 5–15 min at room temperature. These sections were viewed and captured as digital images using a VHX-5000 Digital Microscope (KEYENCE Corporation, Osaka, Japan) at an objective magnification of 40×. A minimum of 3 fibers or 600 cells was counted in each sample.

### Terminal deoxynucleotidyl transferase biotin-dUTP nick end labeling (TUNEL) staining

DNA fragmentation induced by apoptosis was determinated by double-labeled fluorometric TUNEL detection. In brief, 10-μm thick frozen muscle cross-sections were air dried, and fixed in 4% paraformaldehyde in PBS, pH 7.4, at room temperature for 20 min. Then the sections were permeabilized with 0.2% Triton X-100 in 0.1% sodium citrate at 4 °C for 2 min, and incubated with an anti-laminin rabbit polyclonal antibody (1:500; Boster) at 4 °C overnight. After washing with PBS for 30 min, the sections were incubated with the fluorochrome-conjugated secondary antibodies (Thermo Fisher Scientific, Rockford, IL, USA) at room temperature for 2 h. Subsequently, TUNEL (Roche Applied Science, Indianapolis, USA) reaction mix was added in a recommended 1:9 ratio, and the sections were incubated for 60 min at 37 °C in a humidified chamber in dark according to the manufacturer’s protocol. In the end, the section was counterstained with 4′6′ -diamidino-2-phenylindole (DAPI). Moreover, positive and negative controls were included in each experiment. Sections were treated with DNase I (Tiangen, Beijing, China) in DNase buffer for 10 min at room temperature before incubated with the anti-laminin rabbit polyclonal antibody as positive controls, and were incubated without the TdT enzyme as negative controls. Images were visualized using a confocal laser scanning microscope (Olympus, Osaka, Japan) at an objective magnification of 40× and were counted on at least 3 different fields or 600 cells of each sample.

### Quantitative real-time PCR

Total RNAs were routinely extracted from muscles by the RNAiso Plus kit (TaKaRa, Dalian, China) according to the manufacturer’s protocol. RNA quality was determined by measuring the ratio of OD_260_/OD_280_; only samples with OD_260_/OD_280_ > 1.8 were reversely transcribed into cDNA using a TAKARA reagent (TaKaRa, Dalian, China), then stored at −20 °C for subsequent reactions. Quantitative real-time PCR (RT-PCR) was performed using the SYBR Premix Ex Taq II kit (TaKaRa Biotechnology, Dalian, China). Dissolution curves and amplification curves both were firstly observed and the right curve was chosen, a reference gene was *gapdh* and then 2^−△△ct^ method was used to analyze the relative concentration of *bax* and *bcl-2 *mRNA. The primers for RT-PCR were as follows (Sangon, Nanjing, China):

*bax* forward: 5′-GTGGTTGCCCTCTTCTACTTTG-3′,

reverse: 5′-CACAAAGATGGTCACTGTCTGC-3′;

*bcl-2* forward: 5′-GGGATGCCTTTGTGGAACTAT-3′,

reverse: 5′-AGGTATGCACCCAGAGTGATG-3′;

*gapdh* forward: 5′-GACAACTTTGGCATCGTGGA-3′,

reverse: 5′-ATGCAGGGATGATGTTCTGG-3′.

### Western blots

Western blot evaluations were undertaken as previously described^32^. Total protein was extracted from the SOL, EDL and GAS muscles of ground squirrels and solubilized in a sample buffer (100 mM Tris, pH 6.8, 5% 2-β-mercaptoethanol, 5% glycerol, 4% SDS, and bromophenol blue), with muscle protein extracts resolved by SDS-PAGE using Laemmli gels (10% gel with an acrylamide/bisacrylamide ratio of 37.5:1 for Bcl-2 and GAPDH; and 12% gel with an acrylamide/bisacrylamide ratio of 29:1 for Bax and cytochrome C. After electrophoresis, the proteins were electrically transferred to PVDF membranes (0.45μm pore size) using a Bio-Rad semi-dry transfer apparatus. The blotted membranes were blocked with 1% BSA in Tris-buffered saline (TBS; 150 mM NaCl, 50 mM Tris–HCl, pH 7.5) and incubated with rabbit anti-Bcl-2 (50E3) mAb, rabbit anti-Bax large subunit and rabbit anti-cytochrome C (1:1000, Cell Signaling Technology CST, Danvers, MA, USA) and rabbit anti-GAPDH (1:7500, Santa Cruz Biotechnology, Santa Cruz, CA, USA) in TBS containing 0.1% BSA at 4 °C overnight. The membranes were then incubated with IRDye 800 CW goat-anti rabbit secondary antibodies (1:5000) for 90 min at room temperature, and visualized with an Odyssey scanner (LI-COR Biosciences, Lincoln, NE, USA). Quantification analysis of the blots was performed using NIH Image J software.

### Statistical analyses

A one-way ANOVA with Fisher’s LSD post hoc test was used to determine group differences, and the ANOVA–Dunnett’s T3 method was used when no homogeneity was detected. SPSS 19.0 was used for all statistical tests. Statistical significance was accepted for all tests at *P* < 0.05.

## Additional Information

**How to cite this article**: Fu, W. *et al*. Remarkable preservation of Ca^2+^ homeostasis and inhibition of apoptosis contribute to anti-muscle atrophy effect in hibernating Daurian ground squirrels. *Sci. Rep.* **6**, 27020; doi: 10.1038/srep27020 (2016).

## Figures and Tables

**Table 1:** Effects of hibernation on body weight (BW), muscle wet weight (MWW) and ratio of MWW/BW in Daurian ground squirrels.

Group	BW before hibernation (g)	BW at experiment time (g)	MWW at experiment time (mg)			MWW/BW at experiment time (mg/g)		
			SOL	GAS	EDL	SOL	GAS	EDL
PRE	311.86 ± 25.07	311.86 ± 25.07	121 ± 21	255 ± 49	132 ± 25	0.39 ± 0.05	0.81 ± 0.11	0.42±0.06
HIB	304.71 ± 21.31	219.39 ± 30.35***	114 ± 18	221 ± 49	121 ± 20	0.52 ± 0.03**	1.01 ± 0.09*	0.56 ± 0.07**
IBA	314.07 ± 27.31	212.47 ± 29.76***	115 ± 19	218 ± 34	115 ± 16	0.54 ± 0.04**	1.03 ± 0.08**	0.54 ± 0.05**
POST	328.57 ± 33.77	216.48 ± 31.26***	110 ± 18	207 ± 26*	111 ± 22	0.51 ± 0.03**	0.96 ± 0.04	0.54 ± 0.05**

SOL, soleus; GAS, gastrocnemius; EDL, extensor digitorum longus; PRE, pre-hibernation; HIB, hibernation; IBA, interbout arousals; POST, post-hibernation. Data represent mean ± SD; n = 8. ^*^P < 0.05 compared with PRE; ^**^P < 0.01 compared with PRE; ^***^P < 0.001 compared with PRE.
